# Hepatitis C virus infection is an independent prognostic factor in follicular lymphoma

**DOI:** 10.18632/oncotarget.23138

**Published:** 2017-12-11

**Authors:** Joji Shimono, Hiroaki Miyoshi, Takeharu Kato, Takeshi Sugio, Kohta Miyawaki, Tomohiko Kamimura, Takuto Miyagishima, Tetsuya Eto, Yoshitaka Imaizumi, Koji Kato, Koji Nagafuji, Koichi Akashi, Masao Seto, Takanori Teshima, Koichi Ohshima

**Affiliations:** ^1^ Department of Pathology, Kurume University, School of Medicine, Kurume, Japan; ^2^ Department of Hematology, Sasebo City General Hospital, Sasebo, Japan; ^3^ Department of Medicine and Biosystemic Science, Kyushu University Faculty of Medicine, Fukuoka, Japan; ^4^ Department of Hematology, Hara Sanshin Hospital, Fukuoka, Japan; ^5^ Department of Hematology, Kushiro Rosai Hospital, Kushiro, Japan; ^6^ Department of Hematology, Hamanomachi Hospital, Fukuoka, Japan; ^7^ Department of Hematology, Atomic Bomb Disease and Hibakusha Medicine Unit, Atomic Bomb Disease Institute, Nagasaki University, Nagasaki, Japan; ^8^ Department of Hematology, Kurume University, School of Medicine, Kurume, Japan; ^9^ Department of Hematology, Hokkaido University Faculty of Medicine, Sapporo, Japan

**Keywords:** follicular lymphoma, hepatitis C virus, poor prognosis, NS3, overall survival

## Abstract

Hepatitis C virus (HCV) is a single-stranded RNA virus that not only affects hepatocytes, by B cells as well. It is thought that HCV is involved in the onset of B-cell lymphoma. The clinicopathological characteristics of HCV-positive diffuse large B-cell lymphoma (DLBCL) and HCV-positive splenic marginal zone lymphoma (SMZL) are known, but there has been no report on HCV-positive follicular lymphoma (FL). In this study, the clinicopathological characteristics of HCV-positive FL were examined in 263 patients with FL who were classified into a HCV-positive group with HCV antibody and negative groups without one. The number of patients with HCV-positive FL and HCV-negative FL was 10 (3.8%) and 253 (96.2%), respectively. The patients with HCV-positive FL commonly had more than one region of lymphadenopathy, Ann Arbor stage III/IV, hemoglobin <120 g/l, elevated lactate dehydrogenase level, and high-risk categorization of Follicular Lymphoma International Prognostic Index (FLIPI) than in patients with HCV-negative FL. Overall survival and progression-free survival were poorer in patients with HCV-positive FL than in those with HCV-negative FL (*p* < 0.0001 and 0.006, respectively). Also, multivariate analysis revealed that positive HCV antibody was a poor prognostic factor of OS. In conclusion, HCV-positive FL has unique clinical features and may have a great impact on the overall survival of affected patients.

## INTRODUCTION

Follicular lymphoma (FL) is a low-grade lymphoma that is malignant and runs a relatively indolent clinical course. It is said that the median age of onset of FL is the 60 s and is more common in women. FL commonly involves the lymph nodes, but bone marrow infiltration is found in 40–70% of affected patients [1]. The hallmark of this malignancy is a translocation of *BCL2* and *IGH* genes. The identification of this translocation has been implemented as a useful diagnostic method for the detection of lymphomagenesis in patients with FL [1]. The rate of translocation has been reported to be 85–90%.

The hepatitis C virus (HCV) infects some 1.6 million people worldwide. It is an enveloped single-stranded RNA virus that has 1–6 genotypes, shows regional variation [2], and has been associated with chronic hepatitis, liver cirrhosis, and hepatocellular carcinoma (HCC). HCV infection has also been associated with extrahepatic lesions such as malignant lymphoma, cryoglobulinemia, chronic kidney disease, lichen planus, and porphyria [3].

In an epidemiological study of HCV-positive patients with malignant lymphoma, HCV infection was associated with a higher incidence of B-cell lymphoma compared to healthy controls (odds ratio [OR], 10.8) [4]. It has been reported that a high incidence of B-cell non-Hodgkin lymphoma (B-NHL) may be caused by HCV infection, diffuse large B-cell lymphoma (DLBCL; OR, 2.24), marginal zone lymphoma (MZL; OR, 2.47), and lymphoplasmacytic lymphoma (LPL; OR, 2.57) [5]. Meanwhile, there is a report showing the lack of association between FL and HCV infection (OR 0.50), whereas several other reports suggest that FL may be associated with HCV (OR 2.46 and 4.10) [6, 7]. Thus, the association between HCV and FL is still controversial.

It has been shown that there is a variation in the clinical presentation of B-cell lymphoma depending on the presence or absence of HCV. The clinical manifestations of HCV-positive splenic marginal zone lymphoma (SMZL) have been reported to be more common in women; nodular infiltrates and B symptoms are less common [8]. On the other hand, it has been reported that HCV-positive DLBCL is characterized by the common involvement of the spleen and liver [9]. Also, it is more common in the elderly and B symptoms are common; it is also characterized by elevated lactate dehydrogenase (LDH) levels, poor international prognostic index, and involves more than one extranodal site [10–13]. There is a higher incidence of liver damage during chemotherapy, although there is no difference in the overall survival (OS) and progression-free survival (PFS) of DLBCL [12].

Specifically, while the characteristics of HCV-positive SMZL [8] and HCV-positive DLBCL [10–13] have been reported, there has been no report detailing the clinicopathological characteristics of patients with HCV- positive FL. This study analyzes the clinicopathological characteristics of patients with FL based on HCV status.

## RESULTS

### Characteristics of HCV-positive FL

Supplementary Table 1 shows the clinicopathological characteristics of HCV-positive FL. Ten patients were found to be HCV-positive and consisted of 6 men (60.0%) and 4 women (40.0%). The median age was 71.5 years (range, 49–87 years). At initial presentation, 4/10 patients (40.0%) showed B symptoms. No hepatic infiltration was found, and splenic infiltration was seen in only 1 patient (10.0%). Extranodal involvement was found to involve the bone marrow (40.0%, 4/10), gastrointestinal tract (20.0%, 2/10), and skin (10.0%, 1/10). Regarding the Ann Arbor staging system, almost of all of the patients were found to be in stage III/IV and 9/10 patients (90.0%) were categorized as high risk on the Follicular Lymphoma International Prognostic Index (FLIPI). Regarding the histological grade, 7/10 patients (70.0%) showed grade 1/2 and 3/10 patients (30.0%) showed grade 3A. As treatment, 7 patients received rituximab/cyclophosphamide/doxorubicin/vincristine/prednisolone (R-CHOP) therapy and 1 patient received rituximab/cyclophosphamide/vincristine/prednisolone (R-CVP) therapy. Relapse or disease progression was found in 3 patients. Seven patients (70%) died: 3 from lymphoma, 3 from a tumor aside from lymphoma (pancreatic cancer, lung cancer, and langerhans cell sarcoma), and 1 patient died of hepatic failure. The results of immunostaining were as follows: 9/10 (90%) patients showed positive results for CD10, 9/10 (90%) for BCL2, 7/10 (70%) for BCL6, and no patient (0%) for MUM1. Seven patients (70%) tested positive for *IGH-BCL2* translocation, but the frequency did not change in HCV-positive FL as compared with that in HCV-negative FL (70.0% vs. 71.9%, respectively, *p* = 1.00). The incidence of other cancers excluding lymphoma was 9.1% (23/253) for HCV-positive FL and 30.0% (3/10) for HCV-negative FL, with a tendency (*p* = 0.06) to be higher in patients with HCV-positive FL.

### HCV and liver status in HCV-positive FL

**Figure 1:**
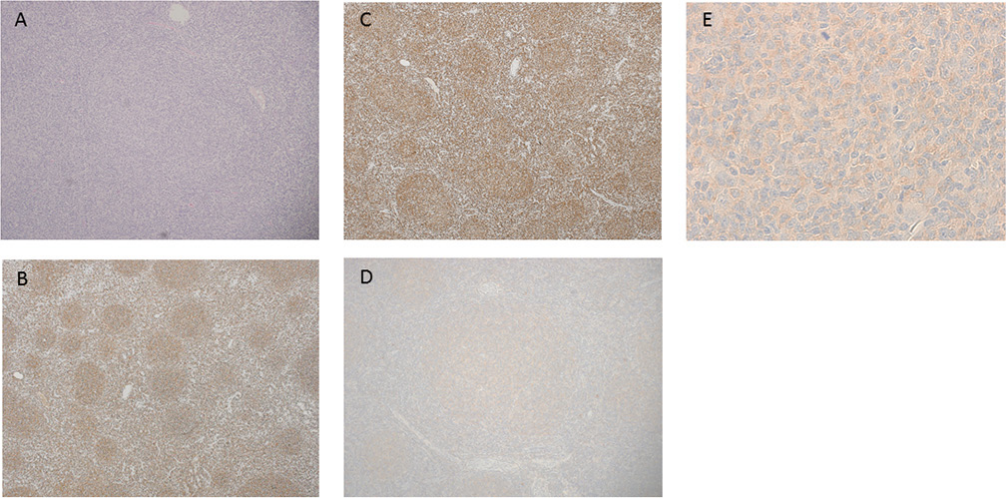
Histological findings in HCV-positive follicular lymphoma (**A**) Hematoxylin-Eosin (HE) staining (×40). (**B**) Immunohistochemical staining of CD20. Tumor cells in neoplastic follicle were positive for CD20 (×40). (**C**) Immunohistochemical staining of *BCL2*. Tumor cells in neoplastic follicle were positive for *BCL2* (×40). (**D**) NS3 positive staining is observed in the neoplastic follicle (×100). (**E**) Tumor cells were positive for NS3 in the cytoplasm (×600).

Table 1 shows the results associated with HCV-positive FL. HCV genotype 1b was found in 6/7 (85.7%) patients, and genotype 2a was found in 1/7 (14.3%) patient. The amount of HCV-RNA present was 2.6–6.7 log IU/ml, and 6/8 (75.0%) patients showed a high viral load (amount of RNA >5.0 log IU/ml). Coinfection with hepatitis B virus was detected in 2/10 patients (20.0%). Regarding liver status, 8/10 patients (80.0%) developed chronic hepatitis, 1/10 (10.0%) patient developed HCC, and 2/10 (20.0%) patients developed cirrhosis. Regarding hepatic toxicity during treatment, there were five cases of Grade 1 and three cases of Grade 2. No patient developed cryoglobulinemia, and 2/10 (20.0%) patients tested NS3-positive. Figure 1 shows the histological findings in HCV positive follicular lymphoma. Regarding HCV eradication therapy, two (case 4 and 8) and 1 patient (case 5) underwent eradication therapy before the onset of FL and after treatment of FL, respectively. The rest of the patients did not undergo eradication therapy. The group receiving eradication therapy tended to have better overall survival and progression free survival than those not receiving eradication therapy, although the difference was not statistically significant.

**Table 1:** Virus and liver status for HCV positive follicular lymphoma in 10 cases

Case	sex	Age(y)	HCV antibody	HCV genotype and viral load		HBV status	Hepatic toxicity	Liver status	Cryoglobulinemia	NS3 immunostaining	HCV eradication therapy
				Genotype	Virus load (log IU/ml)		during treatment				
**1**	M	49	+	2	3.5	**HBc Ab**	NA	LC	−	−	−
**2**	M	75	+	NA	ND	−	Grade 1	CH	−	+	−
**3**	F	64	+	1b	5.6	−	Grade 2	CH	−	−	−
**4**	M	74	+	1b	6.1	−	NA	CH/HCC	−	+	+(Before diagnosis of FL)
**5**	F	52	+	1b	6.7	−	Grade 2	LC	−	−	+(After diagnosis of FL)
**6**	M	77	+	1b	6.4	−	Grade 1	CH	−	−	−
**7**	F	64	+	NA	2.6	HBs Ag	Grade 2	CH	−	−	−
**8**	F	60	+	1b	ND (SVR)	−	Grade 1	CH	−	−	+(Before diagnosis of FL)
**9**	M	78	+	NA	5.5	−	Grade 1	CH	−	−	−
**10**	M	87	+	1b	6.4	−	Grade 1	CH	−	−	−

Abbreviations: M; Male, F; Female, NA; Not available, ND; Not detectable, LC; Liver cirrhosis, CH; Chronic hepatitis, HCC; Hepatocellular carcinoma.

### Clinicopathological comparison of HCV-positive and -negative FL

Table 2 reveals comparison between HCV-positive and negative FL. The patients with HCV-positive FL had a higher frequency of lymphadenopathy in four or more regions (*p* = 0.02), Ann Arbor stage III/IV (*p* = 0.002), hemoglobin <120 g/l (*p* = 0.02), elevated LDH level (*p* = 0.002), and categorization as high risk cases on FLIPI (*p* = 0.0007) than those of HCV-negative FL. There was no relationship with human T-cell leukemia virus type 1 (HTLV-1), human immunodeficiency virus (HIV), or hepatitis B virus (HBV). There was no difference in treatment and complete remission (CR) rate to initial therapy between the two groups.

**Table 2:** Clinicopathological findings between HCV-positive and -negative follicular lymphoma

	HCV positive case (*N* = 10)	HCV negative cases (*N* = 253)	*p*-value
**Age >60 years**	80.0% (8/10)	55.3% (140/253)	0.19
**Male/Female**	6/4	130/123	0.75
**Histological grade**			
**Grade1/2**	60.0% (6/10)	59.3% (150/253)	0.88
**Grade3A**	40.0% (4/10)	33.2% (84/253)	
**Grade3B**	0% (0/10)	7.5% (19/253)	
**Bulky mass**	10.0% (1/10)	9.1%(23/253)	1.00
**Lymphnode >4 regions**	90.0% (9/10)	49.4% (125/253)	0.02
**Bone marrow infiltration**	40.0% (4/10)	27.9% (68/244)	0.48
**Peripheral blood infiltration**	0% (0/10)	6.0% (15/250)	1.00
**Liver infiltration**	0% (0/10)	4.0% (10/253)	1.00
**Spleen infiltration**	10.0% (1/10)	14.6% (37/253)	1.00
**Extranodal infiltration >1**	20.0% (2/10)	7.5% (18/253)	0.17
**B symptoms**	30.0% (3/10)	10.7% (27/253)	0.09
**ECOG performance status >1**	10.0% (1/10)	10.4% (26/249)	1.00
**Ann Arbor Stage III/IV**	100% (10/10)	70.2% (177/252)	0.07
**Hemoglobin <120 g/l**	60.0% (6/10)	23.6% (59/250)	0.007
**Elevated LDH level**	70.0% (7/10)	21.5% (53/247)	0.002
**Soluble IL2 receptor >519 U/ml**	87.5% (7/8)	69.9% (165/236)	0.44
**FLIPI, high risk**	90.0% (9/10)	35.0% (85/243)	0.0007
**Histological transformation**	10.0% (1/10)	3.1% (5/163)	0.30
**HBV infection**	20.0% (2/10)	16.4% (41/250)	0.67
**HIV infection**	0% (0/9)	0% (0/234)	1.00
**HTLV-1 infection**	0% (0/9)	3.4% (8/236)	1.00
**CD10 expression**	80.0% (8/10)	84.9% (203/239)	0.65
**BCL2 expression**	90.0% (9/10)	92.1% (220/239)	0.74
**BCL6 expression**	80.0% (8/10)	95.3% (227/238)	0.07
**MUM1 expression**	10.0% (1/10)	12.6% (30/238)	0.26
***IGH-BCL2*** **translocation**	70.0% (7/10)	71.9% (182/253)	1.00
**Initial therapy**			
**Chemotherapy**	80.0% (8/10)	86.2% (218/253)	0.64
**R-containing regime**			
**R-CHOP**	87.5% (7/8)	91.1% (194/213)	0.54
**R-CVP**	12.5% (1/8)	5.6% (12/213)	
**RTX monotherapy**	0% (0/8)	3.3% (7/213)	
**Others**	0% (0/8)	2.3% (5/218)	1.00
**Radiation therapy**	0% (0/10)	9.5% (24/253)	0.61
**Radiation therapy only**	0% (0/10)	3.2% (8/253)	1.00
**Chemotherapy plus radiation therapy**	0% (0/8)	7.3% (16/218)	1.00
**Watchful wait**	10.0% (1/10)	10.3% (26/253)	1.00
**CR to initial therapy**	62.5% (5/8)	82.9% (184/222)	0.15

Abbreviations: PS; performance status, LDH; lactate dehydrogenase, FLIPI; Follicular lymphoma international prognostic index, R-containing regimen; Rituximab containing regimen, CR; Complete remission.

### Survival analysis for HCV status

**Figure 2:**
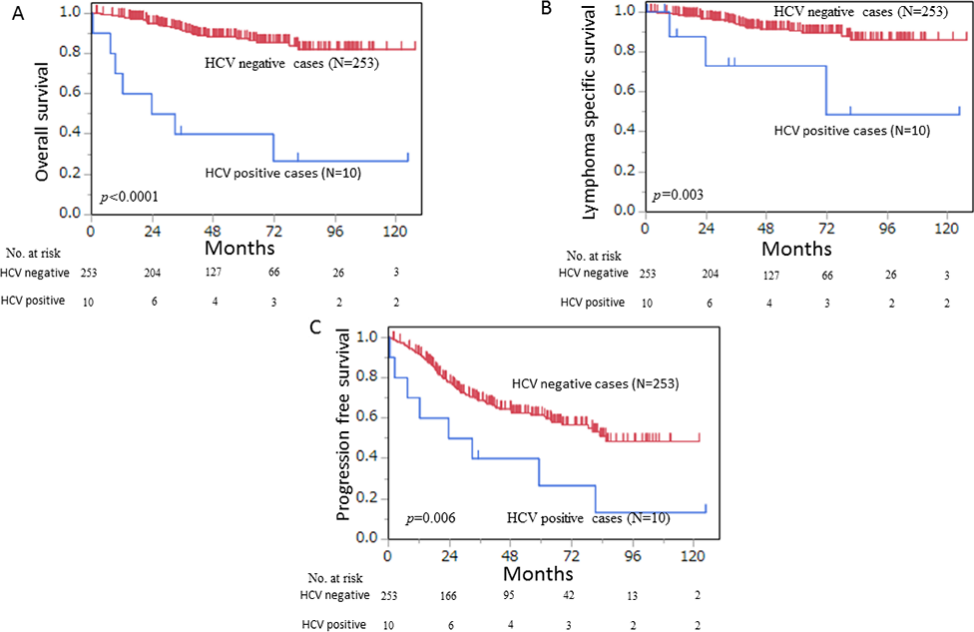
(**A**) HCV-positive follicular lymphoma (*n* = 10) had a significantly worse prognosis than HCV-negative follicular lymphoma (*n* = 253) in overall survival analysis (*p* < 0.0001). (**B**) HCV-positive follicular lymphoma (*n* = 10) had a significantly worse prognosis than HCV-negative follicular lymphoma (*n* = 253) in lymphoma-specific survival analysis (*p* = 0.003). (**C**) HCV-positive follicular lymphoma (*n* = 10) had a significantly worse prognosis than did HCV-negative follicular lymphoma (*n* = 253) in progression-free survival analysis (*p* = 0.006).

The median follow-up periods were 28.3 months (0.5–124.6 months) for patients with HCV-positive FL and 48.1 months (1.9–144.0 months) for those with HCV-negative FL. Figures 2A and 2C show the OS and PFS curves, respectively. With regard to the OS and PFS, the patients with HCV-positive FL showed statistically poor survival compared with that in patients with HCV-negative FL (OS, *p* < 0.0001; PFS, *p* = 0.006). For lymphoma-specific survival (Figure 2B), the patients with HCV-positive FL showed statistically poor survival compared with the patients with HCV-negative FL (*p* = 0.003). When excluding two cases of LC, there was a poor prognosis regarding survival of HCV-positive FL compared with that in HCV-negative FL (*p* < 0.0001, Supplementary Figure 1).

### Univariate and multivariate analysis of OS

Table 3 shows the univariate and multivariate analysis of FL. The results of the analysis were as follows: age >60 years (hazard ratio [HR], 8.19: 95% confidence interval [95% CI], 2.88–23.31: *p* < 0.0001), Eastern Cooperative Oncology Group (ECOG) performance status >1 (HR, 2.35: 95% CI, 1.02–5.40: *p* = 0.04), Ann Arbor stage III/IV (HR, 2.82: 95% CI, 1.10–7.26: *p* = 0.03), hemoglobin <120 g/l (HR, 3.45: 95% CI, 1.77–6.69: *p* = 0.0003), elevated LDH level (HR, 3.36: 95% CI, 1.73–6.53: *p* = 0.0003), and HCV positivity (HR, 3.86: 95% CI, 2.02–7.36: *p* < 0.0001). The results of the multivariate analysis were as follows: age >60 years (HR, 5.36: 95% CI, 1.85–15.51: *p* = 0.002), elevated LDH level (HR, 2.19: 95% CI, 1.03–4.65: *p* = 0.04), and HCV positivity (HR, 5.10: 95% CI, 2.03–12.82: *p* = 0.0005).

**Table 3:** Univariate and multivariate analysis for overall survival in follicular lymphoma

Variable	Overall survival			
	Univariate analysis		Multivariate analysis	
	HR (95% CI)	*p*-value	HR (95% CI)	*p*-value
**Age >60 years**	8.10 (2.40–27.28)	0.0007	5.36 81.85–15.51)	0.002
**Male**	0.80 (0.36–1.78)	0.58		
**Histological grade 3**	1.32 (0.59–2.94)	0.50		
**Bulky mass**	1.92 (0.65–5.61)	0.24		
**Lymphnode >4 reigions**	2.43 (1.01–5.86)	0.04	0.98 (0.40–2.42)	0.97
**Spleen infiltration**	0.87 (0.30–2.53)	0.80		
**Bone marrow infiltration**	1.37 (0.70–2.70)	0.36		
**Liver infiltration**	1.31 (0.18–9.71)	0.79		
**Extranodal infiltration >1**	2.37 (0.70–7.97)	0.16		
**B symptoms**	1.18 (0.35–3.96)	0.79		
**ECOG performance status >1**	3.15 (1.24–7.99)	0.02	1.61 (0.64–4.08)	0.31
**Ann Arbor Stage III/IV**	3.41 (1.02–11.38)	0.04	1.52 (0.48–4.79)	0.48
**Hemoglobin <120 g/l**	2.34 (1.04–5.28)	0.05	1.83 (0.89–3.77)	0.10
**Elevated LDH level**	4.16 (1.86–9.30)	0.0005	2.19 (1.03–4.65)	0.04
**Hepatitis C virus positivity**	5.20 (1.55–17.49)	0.008	5.10 (2.03–12.82)	0.0005
**CD10 expression**	0.96 (0.37–2.50)	0.94		
**BCL2 expression**	3.34 (0.46–24.46)	0.24		
**BCL6 expression**	0.42 (0.15–1.20)	0.10		
**MUM1 expression**	0.52 (0.13–2.09)	0.35		
***IGH-BCL2*** **translocation**	1.59 (0.60–4.23)	0.36		

Abbreviations: HR; Hazard ratio, CI; Confidence interval, LDH; lactate dehydrogenase.

## DISCUSSION

A clinicopathological comparison between HCV-positive and negative FL showed a higher proportion of high-risk categorization for FLIPI and a poorer OS curve was observed in HCV-positive FL than HCV-negative FL. In addition, multivariate analysis revealed that HCV infection was an independent prognostic factor for FL.

In this study, the patients with HCV-positive FL showed a poorer OS than HCV-negative FL. Multivariate analysis also revealed that HCV positive was a poor prognostic factor in patients with FL. So far, there has not been a well-organized report on the clinical characteristics and survival rate in patients with HCV-positive FL. However, in many reports on HCV-positive DLBCL, there was no difference in OS based on the presence or absence of HCV, though the incidence of liver dysfunction during chemotherapy was higher [12, 14, 15]. For SMZL, there is no report that the presence or absence of HCV contributes to OS [8]. The poor OS seen in the patients with HCV-positive FL was attributable to the fact that the number of patients in the high-risk category of FLIPI, which was an existing poor prognostic factor, was significantly higher in the patients with HCV-positive FL than in the HCV-negative group. On the other hand, the number of deaths not attributable to lymphoma was high in this study. The detailed causes of death were as follows: 3 patients died of lymphoma; 3 patients died of neoplastic diseases apart from lymphoma, and 1 patient died of hepatic failure. A relatively high proportion of these patients died from neoplastic disease other than lymphoma. It is considered that the incidence and risk of malignant tumor, except for lymphoma and HCC, in HCV-positive patients is two times higher than HCV-negative patients, which is consistent with the results of our study [16]. Thus it was thought to be one of the determinants of a poor prognosis in patients with HCV-positive FL.

HCV-positive DLBCL with LC is considered to have a poor prognosis compared to that of HCV-positive DLBCL without LC [13]. The reason is that treatment for lymphoma is difficult due to the high incidence of toxicity and impaired liver function for LC [13]. In our study, there were two cases of HCV-positive FL with LC. One case (case 1) was difficult to treat due to liver failure, and the treatment to provide the best supportive care was chosen. In the other case (case 5), R-CHOP therapy was performed, and no recurrence was observed. In addition, the prognosis of HCV-positive FL was poor regarding OS, except for two cases of LC. Owing to the few cases with LC in the present study, the influence of LC on the progression of HCV FL cannot be adequately evaluated. However, the prognosis of HCV-positive FL remained unchanged by the presence or absence of LC.

There have been many reports that HCV eradication therapy, mainly with interferon, led to the reduction or disappearance of lymphoma in HCV-positive B-cell lymphoma, especially the low-grade type. According to a study on HCV eradication therapy in 116 patients with HCV-positive B-cell lymphoma including DLBCL, HCV eradication therapy extends the OS and PFS of patients with HCV-positive B-cell lymphoma. Of the 10 patients with HCV-positive FL in this study, only 1 patient (case 5) underwent HCV eradication therapy after the onset of lymphoma, although eradication therapy was initiated after complete remission after chemotherapy. In our study, we could not sufficiently examine the effectiveness of HCV eradication therapy in HCV positive FL. However, it is possible that the prognosis was better for cases receiving HCV eradication therapy than that in cases not receiving eradication therapy. There is a need to further analyze a larger number of such cases.

*IGH-BCL2* translocation is a translocation found in 70–90% of patients with FL; it is believed to be strongly involved in the lymphomagenesis of FL [1]. *IGH-BCL2* translocation is found in the peripheral blood of healthy subjects and is commonly seen in the elderly [17]. However, since there have been previous reports [18, 19] that lymphocytes in the peripheral blood of HCV-infected patients showed *IGH-BCL2* translocation and disappeared with HCV eradication therapy, it was suspected that there was a possible association between HCV viral load and elevated *IGH-BCL2* translocation in lymphocytes [18]. However, in the current study, the frequency of *IGH-BCL 2* did not change in HCV-positive FL as compared with that in HCV-negative FL. Therefore, this does not support the hypothesis that *IGH-BCL2* translocation occurs due to antigenic stimulation of HCV, resulting in FL.

In conclusion, HCV infection affected the clinicopathological features, including the OS in patients with FL. A more detailed study will be required to determine its effect on lymphomagenesis and to analyze its potential as a therapeutic strategy.

## MATERIALS AND METHODS

### Patients and samples

We investigated 263 patients who underwent biopsy at nine institutions (Karatsu Red Cross Hospital, Kushiro Rosai Hospital, Kyushu University Hospital, Kurume University Hospital, Sasebo City General Hospital, Shimabara Hospital, Nagasaki University Hospital, Hamanomachi Hospital, and Harasanshin Hospital) between 2005 and 2015, and were diagnosed with FL in the Department of Pathology, Kurume University. These 263 patients had been used in our previous reports [20, 21]. All the patients were reviewed by hematopathologists and diagnosed according to the World Health Organization (WHO) classification [1]. Certain subtypes of FL (i.e. pediatric follicular lymphoma, primary cutaneous follicular lymphoma, and primary follicular lymphoma of the duodenum) were excluded from the analyses. The grade of hepatic toxicity during treatment was based on the National Cancer Institute Common Terminology Criteria for Adverse Events (version 4.0). HCV infection was diagnosed in a patient if positive results were observed by using second and third generation immunoassay kits (Monolisa anti-HCV Plus, Sanofi Diagnostics Pasteur, France; and AxSYM HCV Version 3.0, Abbott Laboratories, Parsippany, NJ). The amount of HCV-RNA was measured using quantitative real-time polymerase chain reaction (COBAS^®^ AmpliPrep/COBAS^®^ TaqMan^®^ HCV test; Roche Molecular Systems, Pleasanton, CA, USA), and the genotype was determined using the patients’ serum [22]. HCV-positive FL, except for one case (case 2), was defined based on positive results with the HCV antibody and HCV RNA. Clinical information was obtained by reviewing the patients’ medical charts. The use of materials and clinical information was approved by the Research Ethics Committee of Kurume University, and the study was carried out in accordance with the Declaration of Helsinki.

### Immunohistochemistory analysis

Paraffin-embedded sections of each sample were immunostained. Antibodies (clones) used for immunohistochemistry were CD10 (56C6; Leica Microsystems, Wetzlar, Germany), CD20 (L-26; DakoCytomation, Glostrup, Denmark), BCL2 (124; DakoCytomation), BCL6 (P1F6; Leica Microsystems) and MUM1 (MUM1p; DakoCytomation). Each cases were considered positive if more than approximately 30% of the neoplastic cells were positive.

### Immunostaining of NS3

Immunohistochemical staining of NCL-HCV-NS3 (Leica Microsystems) was carried out by using 2.5-μm-thick, formalin-fixed, paraffin-embedded tissue sections for all cases. The slides were deparaffinized with xylene, followed by ethanol. After rehydration with water, antigen retrieval was performed with EDTA buffer (pH7.0) in a microwave oven at 95°C for 20 minutes. Endogenous peroxidase activity was blocked by incubating in 3% hydrogen peroxide for 5 minutes. Slides were incubated with anti-NS3 mouse monoclonal antibodies (NS3; 1:50 dilution; MMM33, Leica Microsystems) for 30 minutes. The slides were incubated with an EnVision1 System horseradish peroxide-labeled anti-mouse polymer (Dakocytomation) for 30 minutes. Visualization of NS3 was performed using diaminobenzidine for 5 minutes. Slides were counterstained with hematoxylin, dehydrated with ethanol, and mounted under coverslips. Each cases were considered positive if more than approximately 30% of the neoplastic cells were positive.

### Fluorescence *in situ* hybridization (FISH) analysis

FISH was performed to detect *IGH-BCL2* translocation ((KBI-10606, Leica Microsystems) as previously described [23].

### Statistical analysis

The clinicopathological characteristics of the patients were compared by using the Fisher's 2-sided exact test. The end-point of OS was defined as the time of death. The end-point of PFS was defined as the time of relapse due to FL and death. Lymphoma-specific survival was defined as an end point of death due to FL death, excluding deaths from other causes. Survival curves of OS, PFS and lymphoma specific survival were calculated using the Kaplan–Meier method. A log-rank test was used to compare the survival curves. Univariate and multivariate Cox proportional regression models were used to evaluate the proposed prognostic factors. All statistical analyses were carried out using JMP version 11.0 (SAS Institute Inc., Cary, NC, USA), and a value of *p* < 0.05 was considered statistically significant.

## SUPPLEMENTARY MATERIALS FIGURES AND TABLES





## Abbreviations

FL: Follicular lymphoma
HCV: Hepatitis C virus
FLIPI: Follicular lymphoma international prognostic index
OS: Overall survival
PFS: Progression free survival
OR: Odds ratio
SMZL: Splenic marginal zone lymphoma
DLBCL: Diffuse large B-cell lymphoma
LDH: Lactate dehydrogenase
PS: Performance status
LC: Liver cirrhosis
CH: Chronic hepatitis
HCC: Hepatocellular carcinoma.
