# Supplementary material for: Hepatitis C virus infection is an independent prognostic factor in follicular lymphoma
Source: Oncotarget. 2017 Dec 11;9(2):1717–25. doi: 10.18632/oncotarget.23138 (PMC5788593; doi:10.18632/oncotarget.23138)
Supplement: Supplementary file 1 [file oncotarget-09-1717-s001.pdf]

Hepatitis C virus infection is an independent prognostic factor in follicular lymphoma

SUPPLEMENTARY MATERIALS

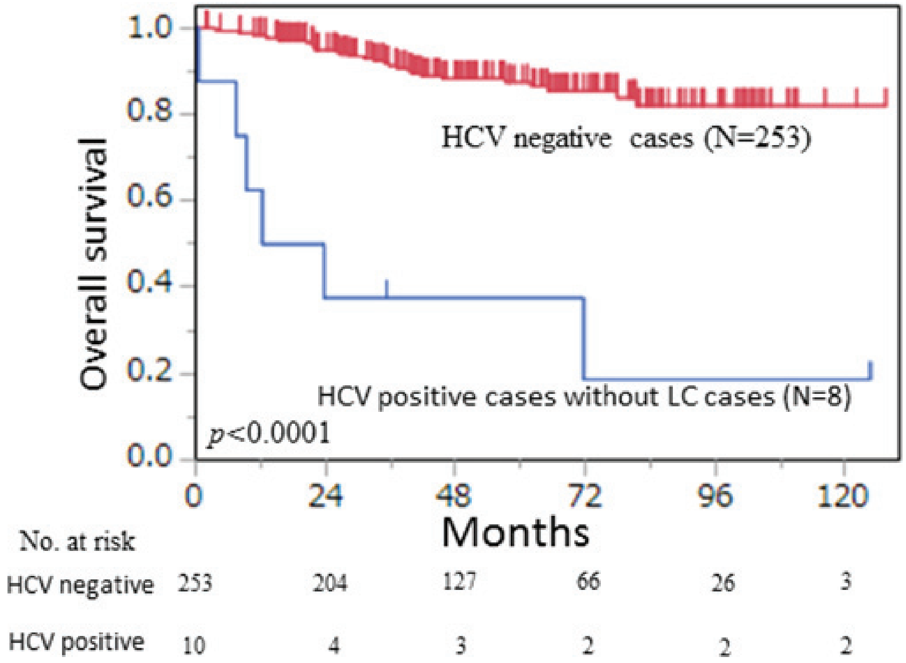

Supplementary Figure 1: Overall survival showed poor prognosis of HCV-positive FL by excluding two cases of LC as compared with HCV-negative FL ($p < 0.0001$).

Supplementary Table 1: Clniccopathological features of HCV positive FL in 10 cases. See Supplementary_Table_1
