# Supplementary material for: Hepatitis C virus infection is an independent prognostic factor in follicular lymphoma
Source: Oncotarget. 2017 Dec 11;9(2):1717–25. doi: 10.18632/oncotarget.23138 (PMC5788593; doi:10.18632/oncotarget.23138)
Supplement: Supplementary file 2 [file oncotarget-09-1717-s002.docx]

Supplementary Table1: Clinicopathological features of HCV positive follicular lymphoma in 10 cases

| **Case** | **Sex** | **Age(y)** | **Primary symptoms** | **Liver infiltration** | **Spleen infiltration** | **Extranodal infiltration** | **Ann Arbor Stage** | **FLIPI, high risk** | **Histological grade** | **Immunohistochemistry** |  |  |  | ***IGH-BCL2* translocation** | **Initial therapy** | **Theraputic effect** | **Relapse** | **Follow up periods (months)** | **Status** |
| --- | --- | --- | --- | --- | --- | --- | --- | --- | --- | --- | --- | --- | --- | --- | --- | --- | --- | --- | --- |
|  |  |  |  |  |  |  |  |  |  | **CD10** | BCL2 | BCL6 | MUM1 |  |  |  |  |  |  |
| **1** | M | 49 | - | - | - | - | III | + | 2 | **+** | + | - | - | + | No therapy | NA | NA | **12** | Dead (Hepatic failure) |
| **2** | M | 75 | B symptoms | - | - | BM, Colon | IV | - | 2 | **+** | + | + | - | + | R-CHOP | CR | - | **33** | Dead (pancreatic cancer) |
| **3** | F | 64 | B symptoms | - | - | Small intestine | IV | + | 2 | **+** | + | + | - | + | R-CVP | PD | + | **9** | Dead (Lymphoma) |
| **4** | M | 74 | - | - | - | BM, Skin | IV | + | 3A | **+** | + | + | - | + | Watchful wait | NA | + | **24** | Dead (Lymphoma) |
| **5** | F | 52 | - | - | + | - | III | + | 1 | **+** | + | - | - | + | R-CHOP | PR | - | **81** | Alive |
| **6** | M | 77 | B symptoms | - | - | - | III | + | 3A | **+** | + | - | - | + | R-CHOP | CR | - | **7** | Dead (Lung cancer) |
| **7** | F | 69 | - | - | - | - | III | + | 3A | **-** | - | + | - | - | R-CHOP | CR | - | **125** | Alive |
| **8** | F | 60 | - | - | - | BM | IV | + | 2 | **+** | + | + | - | + | R-CHOP | CR | - | **35** | Alive |
| **9** | M | 78 | - | - | - | BM | IV | + | 2 | **+** | + | + | - | - | R-CHOP | CR | + | **72** | Dead (Lymphoma) |
| **10** | M | 87 | B symptoms | - | - | - | III | + | 1 | **+** | + | + | - | - | R-CHOP | NA | NA | **0.5** | Dead (Langerhans cell sarcoma) |

M; Male, F; Female, BM; Bone marrow, PB; Peripheral blood, FLIPI; Follicular lymphoma international prognostic index,

R-CHOP; Rituximab, cyclophosphamide, doxorubicin, vincristine and prednisone.

CR; Complete remission, PR; Partial remission, PD; Progressive disease

R-CVP; Rituximab, cyclophosphamide, vincristine and prednisone.
